# Intracranial Mapping of a Cortical Tinnitus System using Residual Inhibition

**DOI:** 10.1016/j.cub.2015.02.075

**Published:** 2015-05-04

**Authors:** William Sedley, Phillip E. Gander, Sukhbinder Kumar, Hiroyuki Oya, Christopher K. Kovach, Kirill V. Nourski, Hiroto Kawasaki, Matthew A. Howard, Timothy D. Griffiths

**Affiliations:** 1 Human Brain Research Laboratory, Department of Neurosurgery, The University of Iowa, Iowa City, IA 52242, USA; 2 Auditory Group, Institute of Neuroscience, Newcastle University, Newcastle upon Tyne, Tyne and Wear NE2 4HH, UK; 3 Wellcome Trust Centre for Neuroimaging, University College London, London WC1N 3BG, UK

## Abstract

Tinnitus can occur when damage to the peripheral auditory system leads to spontaneous brain activity that is interpreted as sound [1, 2]. Many abnormalities of brain activity are associated with tinnitus, but it is unclear how these relate to the phantom sound itself, as opposed to predisposing factors or secondary consequences [3]. Demonstrating “core” tinnitus correlates (processes that are both necessary and sufficient for tinnitus perception) requires high-precision recordings of neural activity combined with a behavioral paradigm in which the perception of tinnitus is manipulated and accurately reported by the subject. This has been previously impossible in animal and human research. Here we present extensive intracranial recordings from an awake, behaving tinnitus patient during short-term modifications in perceived tinnitus loudness after acoustic stimulation (residual inhibition) [4], permitting robust characterization of core tinnitus processes. As anticipated, we observed tinnitus-linked low-frequency (delta) oscillations [5–9], thought to be triggered by low-frequency bursting in the thalamus [10, 11]. Contrary to expectation, these delta changes extended far beyond circumscribed auditory cortical regions to encompass almost all of auditory cortex, plus large parts of temporal, parietal, sensorimotor, and limbic cortex. In discrete auditory, parahippocampal, and inferior parietal “hub” regions [12], these delta oscillations interacted with middle-frequency (alpha) and high-frequency (beta and gamma) activity, resulting in a coherent system of tightly coupled oscillations associated with high-level functions including memory and perception.

## Highlights

• Extensive intracranial recordings were made from an awake, behaving tinnitus patient
• Tinnitus intensity was modulated with tight control over other factors
• Tinnitus is linked to widespread coherent delta-band cortical oscillations
• Rich local cross-frequency interactions link delta to all other frequency bands

## Teaser

Recording from an extensive array of intracranial electrodes in an awake, behaving human patient, Sedley, Gander et al. expose the detailed workings of a brain system responsible for generating tinnitus.

## Results and Discussion

### Subject and Paradigm

**Figure 1:**
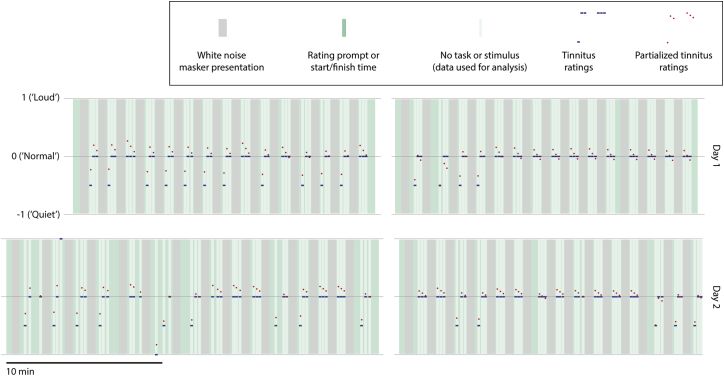
Summary of Experiment The experiment was performed on 2 days (upper and lower rows). On each day, the experiment was divided into two sessions (left and right columns), separated by a short break (gap between columns). Time is denoted by the horizontal axis. In each trial of the experiment, a masker was presented lasting 30 s (gray blocks), followed by four periods in which the subject rated the intensity of his tinnitus (green blocks) that were separated by three silent taskless recording periods of 10 s each (pale green/white blocks) whose data formed the basis of further analysis. Note that the duration of each session was not fixed, but rather depended on the sum of response times (green blocks) across a fixed number of repetitions. The rating scale used consisted of integers from −2 to +2 (though the range of responses given by the subject was −1 to 1, as shown here). A rating of 0 corresponded to the subject’s usual baseline tinnitus intensity, which he confirmed was the same as it had been immediately before the start of each experiment. Each recording period had a tinnitus rating value assigned to it that was the average of the ratings immediately preceding and following that period; these ratings were mainly −0.5 (average of −1, preceding, and 0, following) or 0. Data corresponding to the single rating of +1 (0 pre and +2 post) on day 2 were removed prior to analysis. As tinnitus ratings showed some correlation with overall time elapsed since the start of the experiment and with time elapsed since the end of the preceding masker, the tinnitus ratings were orthogonalized with respect to these variables, yielding partial tinnitus ratings. Tinnitus ratings corresponding to each recording period are shown in blue, and the corresponding partial ratings are shown in red. Note that partial tinnitus ratings have been centered to a mean of zero as part of the partialization process. See also Figure S1 for the subject’s audiological assessment.

The subject was a 50-year-old left-handed male with a typical tinnitus pattern of bilateral tonal tinnitus in association with bilateral hearing loss (Figure S1A). See the Supplemental Experimental Procedures for further subject details and the Supplemental Discussion with regard to the typicality of his case. He underwent chronic intracranial monitoring of the left hemisphere (contralateral to his better hearing ear) for intractable focal seizures. During the invasive monitoring period, his tinnitus was repeatedly transiently suppressed using residual inhibition (RI; transient reduction in tinnitus loudness after presentation of a sound) [4, 8] and assessed by periodic ratings of tinnitus loudness, in between which were 10 s blocks (from which data were used for analysis) in which he was presented no stimuli and had no task to perform. RI can be achieved in the vast majority of tinnitus patients and is thought to suppress tinnitus by temporarily reducing the underlying hyperactivity in the ascending auditory pathway [13]. The subject reported that all masker presentations produced some RI, but there was variation in the duration of the effect across trials. Data collection used for the main analyses commenced after the subject provided his first post-masker rating of tinnitus loudness, and we classified trials as “RI trials” when his tinnitus remained suppressed at this time. The experiment was performed on two separate days, with 15/30 trials on day 1 and 14/30 on day 2 constituting “RI trials.” Efficacy was greater in the first half of each experiment (Figure 1). Given that the task and acoustic stimuli were identical in all trials, the observed results could be considered to represent changes in tinnitus itself, with minimal influence of confounding factors. The presented results were derived from regression of neural activity, recorded electrocorticographically, against the degree of subjective tinnitus suppression. Neurophysiological results were nearly identical across the two experimental days (Figure S2), and the data were therefore pooled for further analysis. Though we recorded mainly from the left hemisphere, we assume that similar results might have been obtained from the right hemisphere as the subject’s tinnitus, the maskers used, and the RI achieved, were all symmetrical.

### Auditory Response Characterization

Evoked, induced, and steady-state auditory responses were measured to 5 kHz (tinnitus-matched) tones and were localized entirely to primary auditory cortex (A1), which was defined physiologically as occupying medial Heschl’s gyrus (HG). See Figure S1B for more details and other stimulus responses.

### Importance of Locally Synchronized Neural Activity and Oscillations

Animal studies measuring correlates of hearing loss and putative behavioral measures of tinnitus have found increased cortical neural synchrony [14] and a possible thalamic origin to this [11]. The equivalent measure in human research is local field potential oscillations, though it is not clear which of the oscillatory frequency bands measured in humans these relate to. Human studies have found alterations in the magnitude of all oscillatory frequency bands in association with tinnitus, particularly delta/theta (1–4/4–8 Hz) [5–7, 9, 15], alpha (8–12 Hz) [6, 15, 16] and gamma (>30 Hz) [5, 8, 15, 17–19].

### Tinnitus-Linked Low-Frequency Oscillatory Power Changes

**Figure 2:**
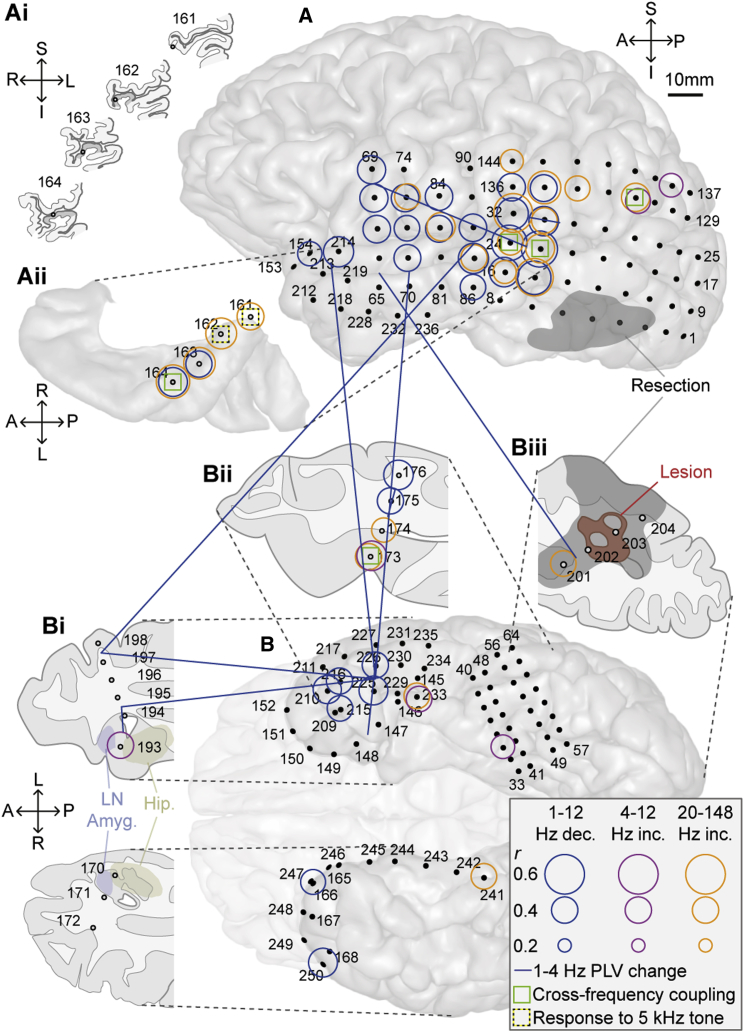
Oscillatory Power and Phase Coherence Changes with Tinnitus Suppression Electrode locations are displayed with respect to a left lateral view of the convexity (A); coronal sections illustrating the location of depth electrode contacts with respect to the gray matter of Heschl’s gyrus (Ai; shown in dark gray); a superior view of the superior temporal plane centered on Heschl’s gyrus (Aii); the inferior surface of the temporal lobes (B); an axial slice through the anterior temporal lobes (Bi); an axial slice through the mid to posterior temporal lobe (Bii), with electrodes 173 and 174 in posterior parahippocampal cortex (PHC); and an axial slice through posterior temporo-occipital cortex, including the epileptogenic lesion (Biii). Axial slices in (Bi), (Bii), and (Biii) are viewed from their inferior aspects (in keeping with the view in B). In (Bi), the solid blue area indicates the lateral nucleus of the amygdala, and the solid yellow area the hippocampus proper, based on an automated segmentation algorithm implemented in FreeSurfer (https://surfer.nmr.mgh.harvard.edu/). In (Biii), the red area represents the lesion, and the gray area (also in A) represents the extent of tissue subsequently resected. Subdural electrodes are represented by solid black circles and depth electrodes by gray filled circles. Significant oscillatory power changes are denoted by colored hollow circles, with circle radius representing the peak correlation, within any of the specified frequency bands, between power and tinnitus *suppression*. Note that neural activity changes are displayed on all sections except (Ai), which is for illustrative purposes only. Blue, magenta, and orange circles indicate delta/theta/alpha (1–12 Hz) decreases, theta/alpha (4–12 Hz) increases, and beta2/gamma (28–148 Hz) increases, respectively. Phase coherence (PLV) changes were found only in the delta (1–4 Hz) band and are represented by solid blue lines. Each PLV connection was calculated between two bipolar electrode pairs (each consisting of two adjacent electrodes), and each end of each line is placed in between the two electrodes comprising that bipolar pair. Green boxes indicate sites where local cross-frequency coupling correlated significantly with tinnitus suppression (see Figure 4). Yellow and black boxes denote electrodes showing induced oscillatory and steady state responses to tinnitus-matched tones (see Figure S1B). See also Figure S2 for a full summary of power changes for both repetitions of the experiment.

**Figure 3:**
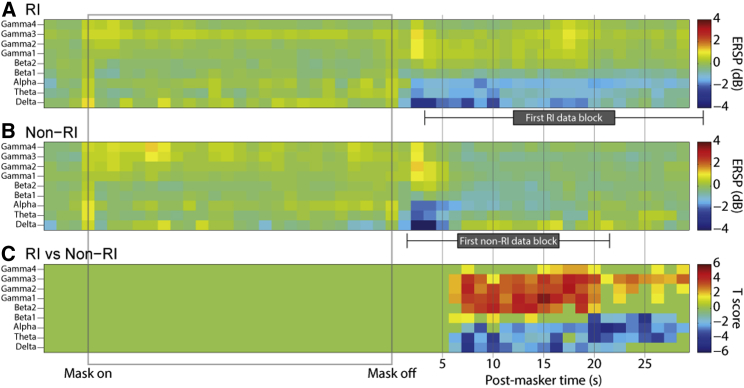
Time-Frequency Decomposition of Oscillatory Power Changes Occurring during and after Noise Masker Presentation The horizontal axis represents time, from 5 s before masker onset, through the 30 s of masker presentation (gray box), and the 30 s following masker offset (during some of which the data constituting the main analyses were captured). Note that the next masker was presented much later than 30 s after the end of the present masker and that these plots simply present the time period during which most tinnitus suppression occurred. Vertical axes represent frequency in the same bands as featured in other analyses and figures. (A) and (B) show mean power changes, across trials, relative to pre-masker baseline (i.e., the 5 s before each masker), expressed as event-related spectral perturbations (ERSPs; 10 times the base-10 logarithm of the power to baseline ratio), grouped according to whether or not the first post-masker tinnitus rating indicated that RI was maintained at that time (RI trials) or had ceased (non-RI trials). Power values shown were averaged across all electrodes in auditory cortex (HG and STG). (C) shows T scores, for each time-frequency point, of the difference between RI and non-RI trials, thresholded for significance with a cluster approach at p < 0.05 corrected. During much of the post-masker period, the subject was engaged in the task of giving ratings of his tinnitus loudness. Periods in which no task was performed (i.e., between one rating and the prompt for the next) were 10 s long, and their position varied depending on response time. The mean positions of these 10 s data blocks for RI and non-RI trials are indicated by gray boxes underneath the respective axis, and their SDs are indicated by horizontal error bars. See also Figure S3 for a trial-by-trial representation of peri-masker power changes.

We predicted that tinnitus suppression would correlate with reduced delta (1–4 Hz) and/or theta (4–8 Hz) oscillatory power, thought to be triggered by the thalamocortical tinnitus input [5–10, 15]. As expected, we observed delta and theta power decreases in auditory cortex (Figure 2). These were present in all auditory cortex sites recorded, except for A1. As the division of the human thalamus implicated in tinnitus (the suprageniculate/limitans complex [10]) projects to A1 and non-primary auditory cortex [20], A1 could potentially be bypassed by thalamic projections that might drive tinnitus. Alternatively, A1 might show elevated activity in tinnitus that does not suppress with RI. We also saw concordant alpha (8–12 Hz) power decreases, which are discussed later. The same pattern of delta/theta/alpha suppression was seen along the full length of superior temporal gyrus (STG), posterior middle temporal gyrus (MTG), inferior temporal pole (TP), inferior parietal cortex (IPC), and primary sensorimotor cortex (S1/M1). In contrast, only A1 responded to presentation of a loud tone, matched to the subject’s tinnitus frequency of 5 kHz. These results are displayed in Figures 2 and S1B. We also examined the time course of oscillatory power changes before, during, and after the presentation of maskers that did and did not result in sustained RI (Figure 3), which shows that these oscillatory power changes occurred rapidly after masker offset, and the difference between RI and non-RI trials was that they lasted longer in RI trials.

### Long-Range Propagation of Pathological Low-Frequency Oscillations

Given the wide anatomical area in which oscillatory power changes were observed, we aimed to establish the pattern of long-range connectivity through which these brain regions interacted. We examined oscillatory phase coherence between all electrode pairs, expressed as phase locking value (PLV) [21]. Significant PLV changes, accompanying tinnitus suppression, were found only in the delta frequency band (Figure 2); these were mainly long range and linked STG with TP, anterior mesial temporal lobe (aMTL), parahippocampal cortex (PHC), and M1. Connections were also found between aMTL and TP.

### Tinnitus-Linked High-Frequency Oscillations

The theory of thalamocortical dysrhythmia proposes that the underlying tinnitus drive, in the form of low-frequency oscillations, causes unbalanced lateral inhibition in cortical “edge” regions between normal areas and those expressing pathological low frequency oscillations, leading to high-frequency gamma oscillations that are (at least in part) the basis of tinnitus perception [5, 22]. While there is support for this theory from resting-state studies, during short-term changes in tinnitus intensity, group-level changes in gamma power have not been found [7, 9], and significant individual-level changes have shown both positive and negative correlations with tinnitus strength [8]. In studies of normal brain function, gamma oscillations have been associated with cognition, attention, perception, and prediction errors [23–25]. It remains unclear which of these processes are associated with tinnitus-related gamma [26], and varying contributions of each could potentially explain the lack of a straightforward association between tinnitus strength and gamma magnitude. We therefore hypothesized that gamma power changes would be seen during tinnitus suppression but that these could take the form of either increases or decreases. A previous PET study using RI found that tinnitus suppression was associated with increased blood flow in MTG and STG [27], which, based on the strong association between local blood flow and gamma oscillations [28], may suggest increased gamma power in RI and/or decreased power in lower frequencies. We observed that, during tinnitus suppression, there were widespread *increases* in both gamma (>28 Hz) and beta2 (20–28 Hz) power. These occurred throughout all of auditory cortex, including A1, and in other cortical areas, including MTG, IPC, S1/M1, and PHC (Figures 2 and S2); they were too extensive and confluent to constitute an “edge effect.” The data suggest instead that these gamma and beta changes are directly related to the pathological delta activity.

### Tinnitus-Linked Alpha Oscillations

A number of studies have shown decreased alpha oscillatory power and variability (8–12 Hz) in auditory regions as a correlate of tinnitus [6, 15, 16, 29], though the opposite has also been seen [30]. Contrary to expectation, we observed *decreased* alpha power with the suppression of tinnitus (i.e., a positive correlation between alpha power and tinnitus strength) in areas showing delta/theta decreases, with only four separate electrodes showing alpha increases (Figures 2 and S2), located in anterior and posterior mesial temporal lobe and IPC. In a study of illusory percepts after the offset of notched noise (Zwicker tones), which may share some underlying mechanisms with tinnitus, a cortical network was identified in which alpha power inversely correlated with the strength of the illusion [31]; this included primary and secondary AC, as well as IPC. In the present study, we found a similar distribution of alpha (along with delta and theta) reduction during tinnitus suppression. While these findings could be considered discordant, in that alpha power has the opposite relationship with perceptual intensity, they can be reconciled if we are to consider alpha as a correlate of deviation from a baseline perceptual state (which could reflect silence or ongoing tinnitus). Alpha oscillation strength also correlates inversely with attention, and therefore changes in attention (top down or stimulus driven) could affect the relationship between tinnitus strength and alpha power. Overall, these findings are not explained by a universal inhibitory model of alpha and corroborate the need for further studies of its role [32].

### Local Cross-frequency Interactions at Cortical “Hub” Regions

**Figure 4:**
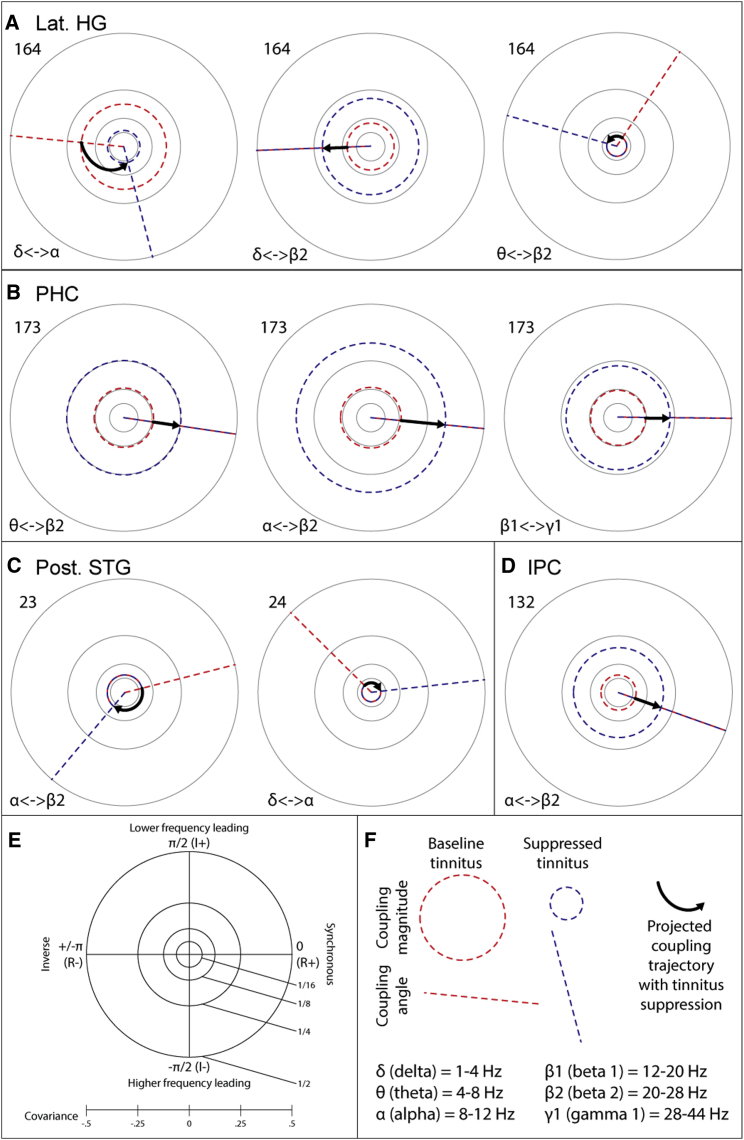
Local Cross-frequency Coupling Changes Coinciding with Tinnitus Suppression Polar plots, in the complex plane, of significant changes in local cross-frequency envelope coupling as a function of tinnitus suppression. The five electrodes featured in this figure are highlighted with green boxes in Figure 2. In each plot (as summarized in E) the horizontal axis represents real-valued (R; non-lagged) coupling, with values to the right of the origin indicating positive coupling and to the left negative or anti-coupling. Real-valued envelope coupling is equivalent to envelope covariance, and thus an intuitive impression of the coupling results can be gained by just looking at the horizontal axis of each plot and ignoring the vertical. The vertical axis represents imaginary (I; phase-lagged) coupling, with values above the origin indicating the lower frequency leading and below the higher frequency leading. Distance from the origin indicates the strength of coupling. In each plot (as illustrated in F), the red dashed circle and line indicate the magnitude and phase difference, respectively, of coupling in the baseline tinnitus state. Blue dashed circles and lines indicate the magnitude and phase difference, respectively, of coupling in the suppressed tinnitus state. These are placed exactly on the equivalent red circles and lines in cases where magnitude or angle does not change significantly. These circles and lines indicate the state of coupling during the maximum partial suppression generally seen during the experiment. Bold arrows indicate the projected path of coupling with increasing tinnitus suppression, based on polar coordinate interpolation between the baseline and suppressed states. The number by each plot indicates the electrode number at which coupling is illustrated, and the Greek letters show which frequency bands the coupling being illustrated is between. HG, Heschl’s gyrus (A); PHC, parahippocampal cortex (B); STG, superior temporal gyrus (C); IPC, inferior parietal cortex (D). See also Figure S4, for a summary of cross-frequency coupling changes in the context of other neural activity changes.

To characterize local interactions between frequency bands, which might allow the various neural processes already discussed to function as a coherent whole [12], we quantified changes in the cross-spectral density between the power envelopes of each pair of frequencies (except adjacent frequency bands) at each electrode. This measure is equivalent to the covariance of the two power envelopes, but it captures additional information, including the phase lag inherent in the coupling. Five electrodes, marked with green boxes in Figure 2, featured a total of nine tinnitus-linked cross-frequency interactions (Figure 4), which in combination included all frequency bands from delta to gamma1 (28–44 Hz). In auditory areas (lateral HG, which is strongly linked to pitch processing [33, 34], and posterior STG), tinnitus suppression accompanied increases in delta-alpha coupling and decreases in high-frequency (beta2) to low-frequency (delta, theta, and alpha) coupling. Previous recordings from monkey V1 have found resting-state anti-correlation (negative covariance) between alpha and high-frequency power [35], generated in deep and superficial cortical layers, respectively. In our subject, baseline tinnitus intensity was associated with the opposite pattern (positive coupling between alpha and higher frequencies), which reversed during tinnitus suppression, perhaps indicating a return to a more “normal” state. In areas linked to auditory memory (IPC and PHC), positive coupling was observed between theta/alpha and higher (beta2 and gamma1) frequencies, which became stronger when tinnitus was suppressed.

### Future Directions of Research

While derived from a single case, the present findings generally concord well, where applicable, to the more reproducible parts of the human tinnitus literature and to those of the one existing human intracranial case study of tinnitus [36]. There remains a need for replication of these findings in other similar cases and, where possible, to examine other cortical areas not presently sampled (such as precuneus, lateral prefrontal cortex, and several divisions of cingulate cortex). While these opportunities are limited in humans, aspects of the present approach could be implemented in animal studies through the use of widespread simultaneous cortical recordings. RI could in principle also be used in animals. The consistent relationship between delta/theta oscillations and tinnitus intensity in this and other studies supports these oscillations as a neural correlate of tinnitus that might be further investigated mechanistically in animal models. The relevance of other frequency bands to tinnitus, however, requires further clarification, and we suggest a possible model below.

### A Working Hypothesis of Interacting Tinnitus Sub-networks

Figure S4 summarizes the tinnitus-linked neural activity changes identified in this study, along with their putative roles with respect to tinnitus. Examination of these findings suggests the possibility of three separate tinnitus “sub-networks,” each manifest in a different frequency band, which interact in specific sites through cross-frequency coupling. One of these sub-networks is characterized by widespread changes in delta (and theta/alpha) power and delta coherence (blue in Figures 2 and S4), thereby representing propagation of the delta-band signal, which is hypothesized to be a consequence of aberrant thalamic input. We thus refer to this as the “tinnitus driving network.” It appears that non-primary auditory cortex in STG has a particularly crucial role in coordinating long-range communication in this network. The second sub-network is tightly localized to areas implicated in auditory memory, namely parahippocampal and inferior parietal cortex [12, 37], and is characterized by increases in alpha (±theta) power (magenta in Figures 2 and S4) during tinnitus suppression that are inverse to changes seen within the tinnitus driving network. We putatively suggest that this network relates to tinnitus-linked mnemonic processes, such as encoding or retrieval of the percept, or comparison to previous auditory memories. We refer to this as the “tinnitus memory network.” Finally, a moderately widespread sub-network is characterized by changes in high-frequency (orange in Figures 2 and S4) beta and gamma power (representing perceptual information processing [23], which may include predictive models and prediction errors [25, 38]). We therefore propose that this sub-network represents the real-time encoding of changes to the tinnitus percept and thus highlights the signatures of neural activity that are the most closely linked to the subjective experience of tinnitus itself. We refer to this as the “tinnitus perception network.” Notably, the sites of cross-frequency interaction (green in Figures 2 and S4) linking these possible sub-networks (auditory cortex, PHC, and IPC) closely resemble the layout of a recently-speculated “tinnitus core” network [12], comprising the minimum neural ensemble required for conscious tinnitus perception. The same model proposes that this kind of local cross-frequency interaction is a fundamental organizing principle of cortical tinnitus networks. Thus, our findings provide direct support for both the anatomical and physiological aspects of this hypothesis and go further in revealing the detailed workings of such a “tinnitus system” through which the low-frequency tinnitus input signal propagates spatially, across the cortex, and spectrally, across frequency bands, so as to lead to a perceived auditory entity.

## Experimental Procedures

While undergoing invasive electrode monitoring, the tinnitus subject participated in identical RI experiments [4, 8], on two separate days, in which he was periodically presented noise maskers in between which he gave periodic ratings of his current tinnitus loudness. Electrocorticography data recorded in between maskers and ratings were compared with contemporaneous tinnitus ratings. The study was approved by the University of Iowa Institutional Review Board, and written informed consent was obtained from the subject prior to any experimentation. The following neural activity metrics were calculated and expressed as a function of tinnitus suppression: (1) oscillatory power at every electrode in each frequency band; (2) long-range phase coherence (phase locking value [21]), between every possible pair of electrodes, in each frequency band; and (3) local cross-frequency coupling, at each electrode, between the power envelopes of every possible pair of frequency bands (except adjacent bands). Statistical analysis was performed with a permutation approach, intrinsically corrected for multiple comparisons, with a significance threshold of p < 0.05. A time-frequency decomposition was also performed within all auditory cortex electrodes, over the peri-masker time period. External auditory stimulation, with tinnitus-matched and other simple stimuli, was also performed, on separate days from the RI experiments. See the Supplemental Experimental Procedures for further details.

## Author Contributions

W.S. and P.E.G. contributed equally to this work and are joint-first authors. The subject was identified and recruited by M.A.H. Clinical and audiological assessment was performed by P.E.G. and T.D.G. Electrode implantation was performed by M.A.H. and H.K. Experiments were designed by W.S., P.E.G., S.K., and T.D.G., run by P.E.G., and coordinated by K.V.N. Electrophysiologic data were analyzed by W.S. with input from P.E.G., C.K.K., and S.K. Anatomical data were processed and illustrated by H.O., C.K.K., and W.S. The manuscript was prepared by W.S. with input from all other authors.

## Supplemental Information

Document S1. Supplemental Discussion, Supplemental Experimental Procedures, and Figures S1–S4

Document S2. Article plus Supplemental Information
