# Supplementary material for: Intracranial Mapping of a Cortical Tinnitus System using Residual Inhibition
Source: Curr Biol. 2015 May 4;25(9):1208–14. doi: 10.1016/j.cub.2015.02.075 (PMC4425458; doi:10.1016/j.cub.2015.02.075)
Supplement: Document S1. Supplemental Discussion, Supplemental Experimental Procedures, and Figures S1–S4 [file mmc1.pdf]

Current Biology

Supplemental Information

Intracranial Mapping of a Cortical Tinnitus System using Residual Inhibition

William Sedley, Phillip E. Gander, Sukhbinder Kumar, Hiroyuki Oya, Christopher K. Kovach, Kirill V. Nourski, Hiroto Kawasaki, Matthew A. Howard III, and Timothy D. Griffiths

Supplemental figures

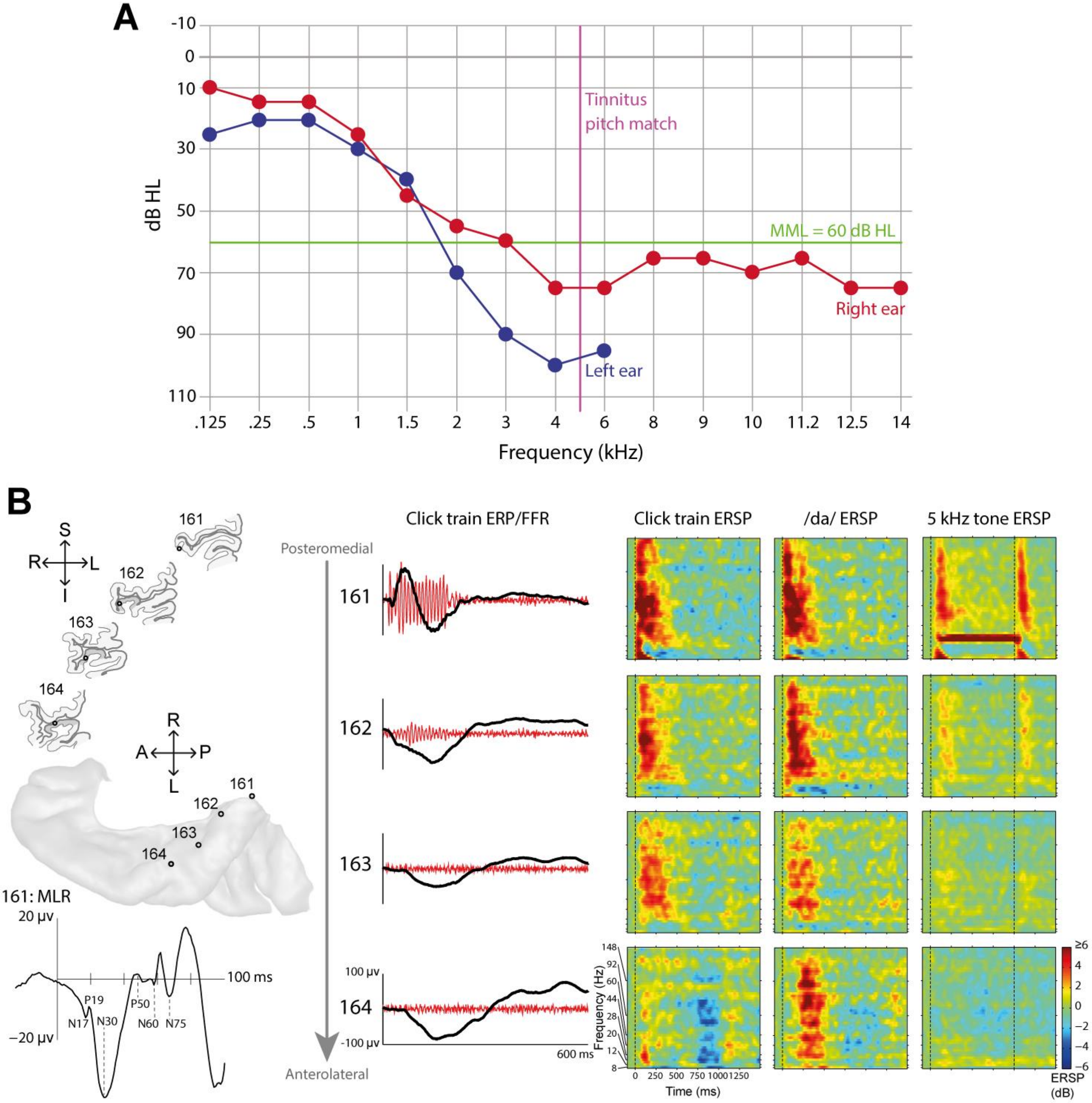

Figure S1, related to Figure 1: Subject's audiometric data and auditory responses

(**A**) Results of the subject's extended pure-tone audiogram, performed in the month prior to the experiment, are shown in blue (left ear) and red (right ear). Testing above 6 kHz in the left ear resulted in no response. The patient described his tinnitus as resembling a pure tone, and matched it to a tone at 5 kHz. The minimum masking level (MML), defined as the quietest level of speech spectrum-shaped noise that abolished the tinnitus percept, was found to be 60 dB HL. (**B**) Responses to broadband and tinnitus-matched auditory stimuli

Upper and middle parts of the left column illustrate the anatomy of Heschl's gyrus (HG) and the position of electrodes within it, as in Figure 2. Below these is the middle latency response (MLR) waveform (trial average of the 10 Hz high-pass filtered signal) to a 25 Hz click train, which gave the clearest response on account of the low

click rate. Waveform peaks are labelled in accordance with previous work examining MLRs in human HG [S31]. The remainder of the figure illustrates the following stimulus responses, in the four electrodes within HG (161 to 164, each row representing one electrode): evoked potentials (ERP; black) and frequency following responses (FFR; red) to 100 Hz click trains (FFR waveforms band-pass filtered around 100 Hz); ERSPs to 100 Hz click trains; ERSPs to /da/ consonant-vowel stimulus; ERSPs to a 1 s 5 kHz tone (matched to tinnitus frequency), which also contains a 40 Hz amplitude modulation that elicits a 40 Hz FFR in electrode 161. Note that the click train ERSP also contains a FFR (at 100 Hz) but, due to the short stimulus duration, this overlaps with the broadband stimulus onset response. Dashed lines on ERSP plots indicate stimulus onset, and for 5 kHz tones the second line indicates stimulus offset. Primary auditory cortex (PAC) is defined physiologically as the part showing FFRs at 100 Hz. By this measure, electrode 161 is in PAC, with 162 being close to its edge. No electrodes are included outside of HG, as none of these showed any definite responses to these stimuli. ERSPs = event-related spectral perturbations, defined as 10 times the base-10 logarithm of the ratio of post-stimulus power to pre-stimulus power (i.e. decibel [dB] ratio).

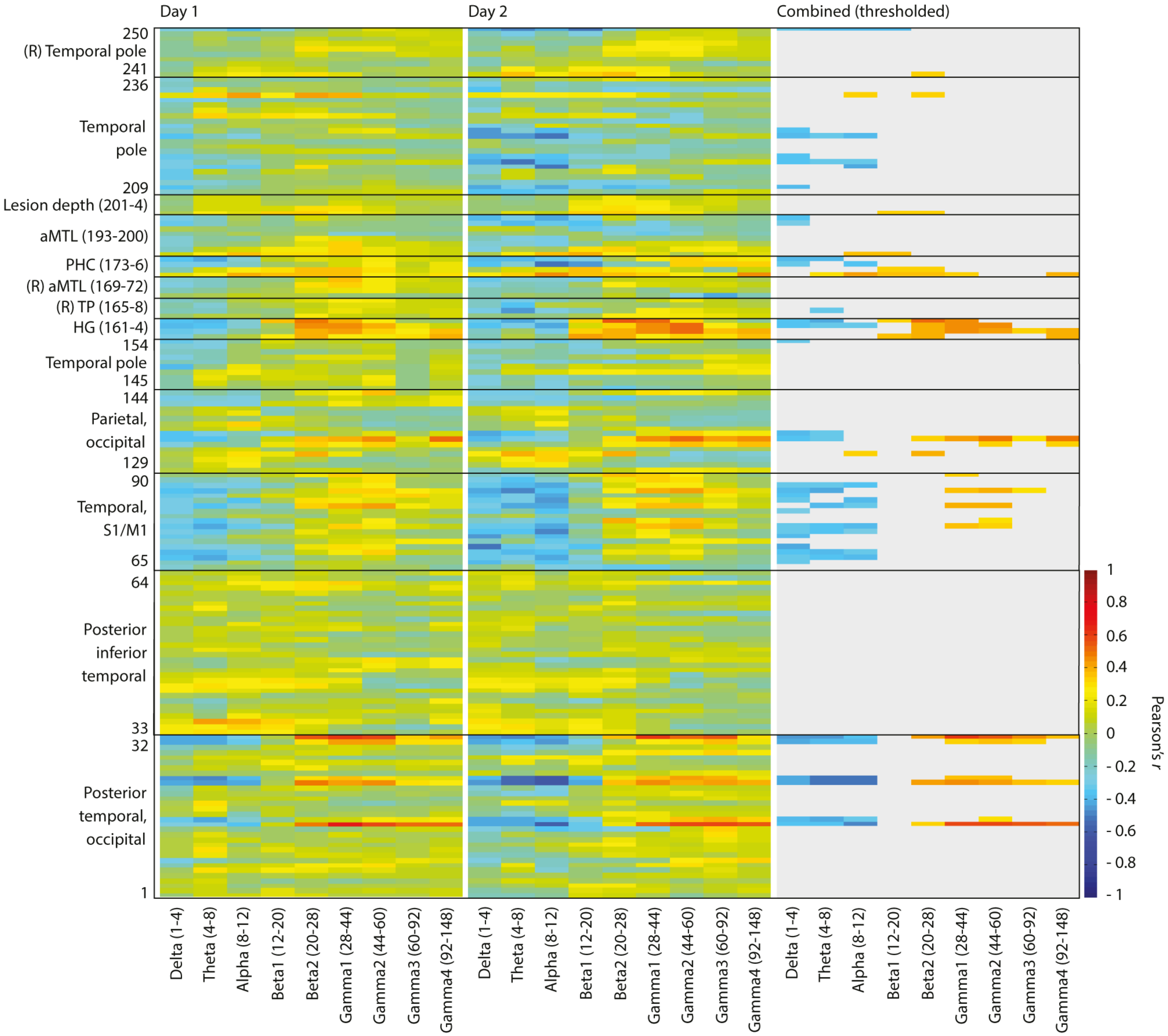

Figure S2, related to Figure 2: Oscillatory power changes correlating with tinnitus suppression

Three data matrices are displayed, corresponding to (left to right) days 1 and 2 of the experiment, and the combined results thresholded at $p < 0.05$ corrected. Rows in the matrices represent individual electrodes (numbers correspond to those in Figure 2) and columns individual frequency bands (bracketed numbers indicate Hz). Color values denote the correlation coefficients (Pearson's *r*) between partial tinnitus suppression and power in any specific electrode/frequency combination. Cool colors indicate power decreases, with tinnitus suppression, and hot colors power increases. In the thresholded plot, gray colors indicate the absence of significant power change. S1/M1 = primary somatosensory/motor cortex, TP = temporal pole, HG = Heschl's gyrus, aMTL = anterior mesial temporal lobe, PHC = parahippocampal cortex.

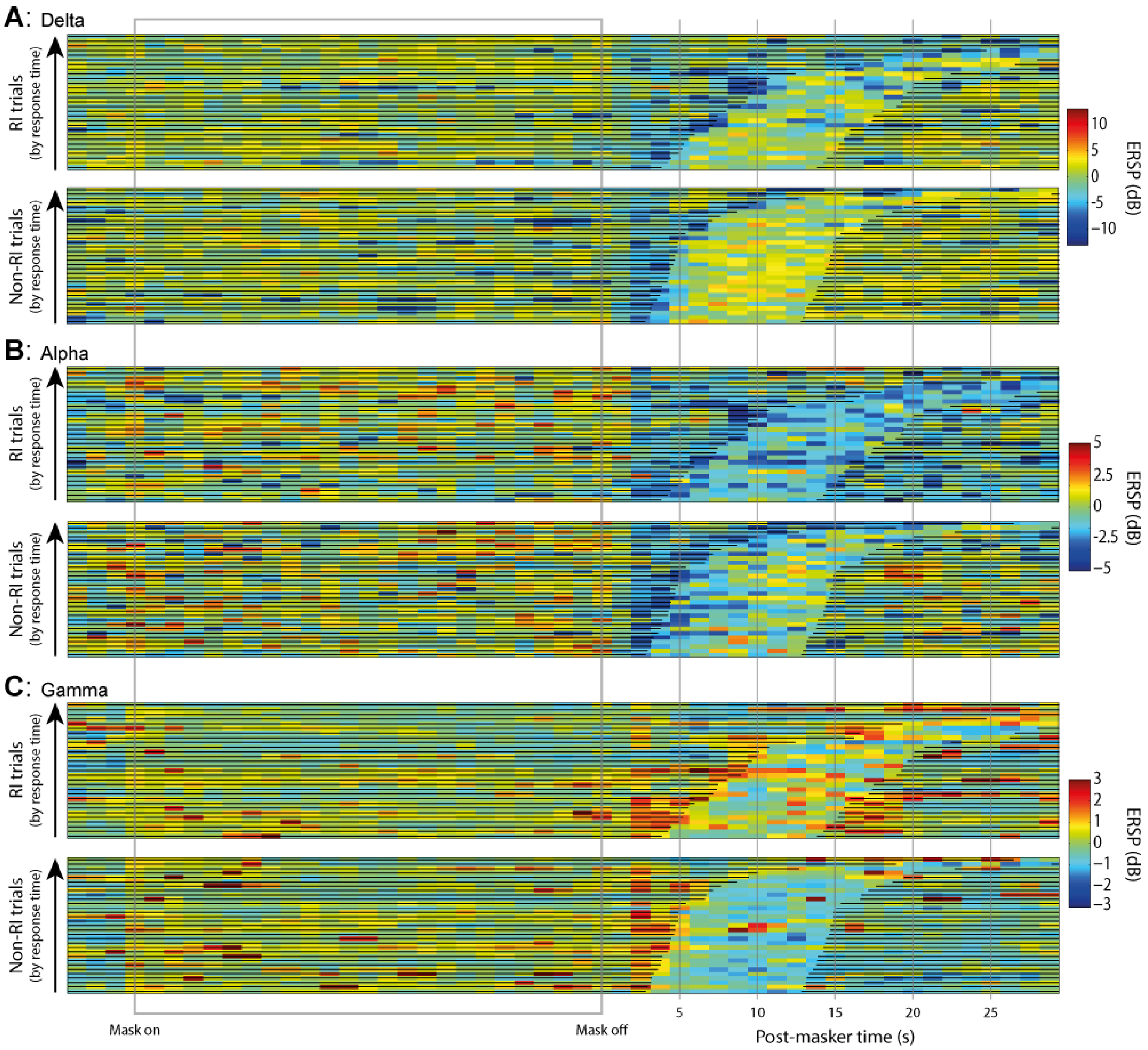

Figure S3, related to Figure 3: Individual trial power changes during and following masker presentation
This figure displays the individual trial breakdown of what is shown in Figure 3, limited to just three frequency bands: delta, alpha and gamma (average of gamma1, gamma2, gamma3 and gamma4). Horizontal axis denotes time, including 5 s before masker onset, the 30 s masker period (gray box), and a subsequent 30 s. Individual trial power values are expressed as ERSPs (see Figure 3 legend for definition), relative to a common baseline calculated from the average of all trials across both RI and non-RI conditions. The vertical axis represents trial, with each row constituting one trial. Trials are sorted (low to high) according to the time taken to give the first tinnitus rating (short to long). Horizontal black lines on the color images indicate time periods where either a masker was playing or the subject was required to give a response. Thus, the sections without lines through represent the 10 s silent, taskless periods that followed the first tinnitus rating, and whose data formed the basis for most of the analyses presented.

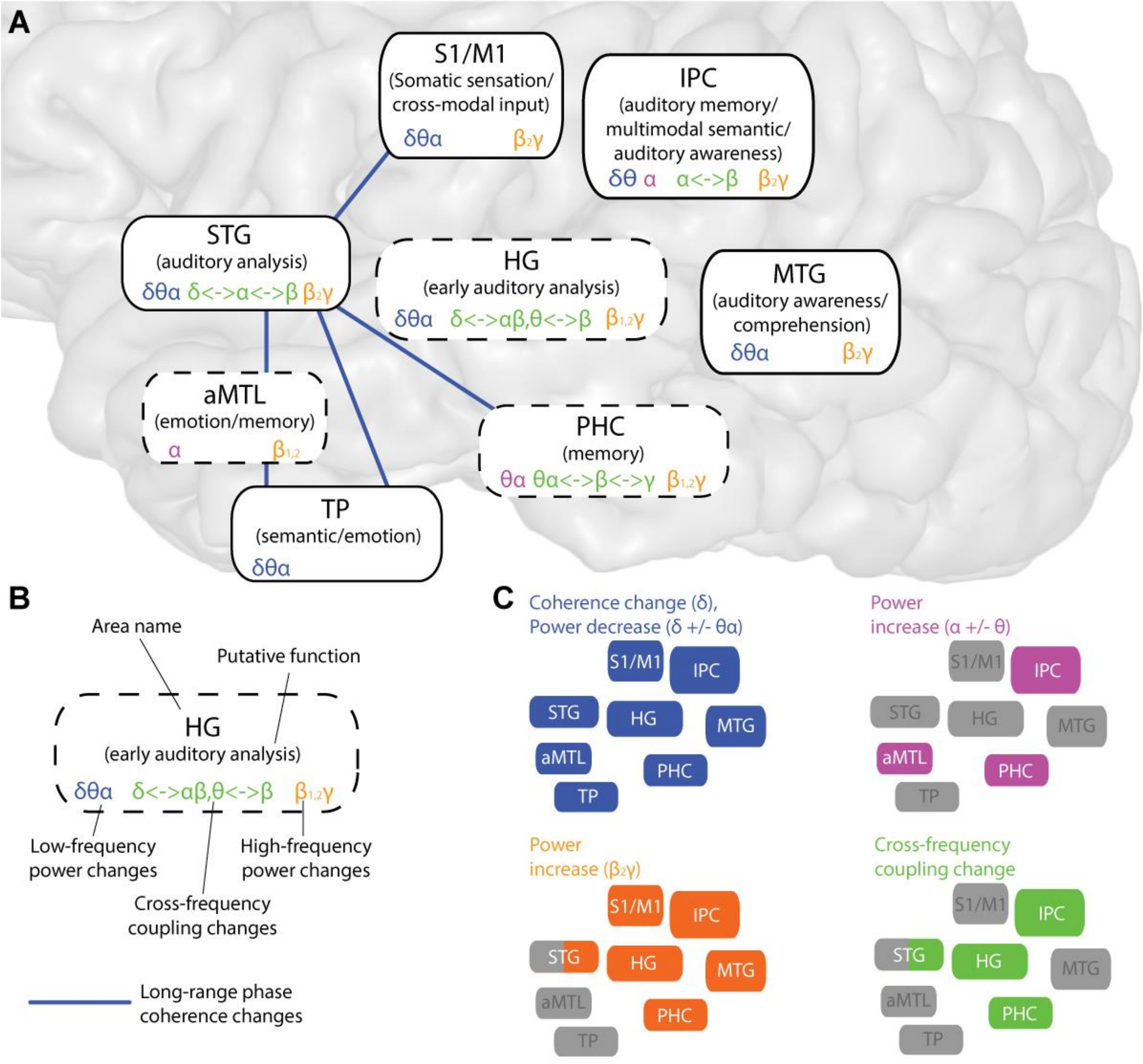

Figure S4, related to Figure 4: Summary of neural activity changes and their putative functions with respect to tinnitus

Panel **A** summarizes the principal empirical observations from this subject's data. Panel **B** indicates the notation used in panel **A**. Panel **C** groups the neural activity changes into four overlapping but anatomically distinct patterns, based empirically on consistent patterns of neural activity changes within each. White text boxes with solid and dashed outlines indicate superficial and deep areas respectively. Each brain area is labelled with its name and its putative function with respect to tinnitus (based on current theory). As illustrated in **A**, Greek letters within the text boxes indicate significant local oscillatory power changes; blue and magenta letters indicate local low-frequency decreases and increases, respectively, occurring as a function of tinnitus suppression, while orange letters indicate high frequency power increases. Blue lines indicate altered long-range delta-band phase coherence with tinnitus suppression. Green Greek letters indicate interaction between these separate modes of activity, in the form of altered local cross-frequency coupling. S1/M1 = primary somatosensory and motor cortex, IPC = inferior parietal cortex, STG = superior temporal gyrus, HG = Heschl's gyrus, MTG = middle temporal gyrus, aMTL = anterior mesial temporal lobe, PHC = parahippocampal cortex, TP = temporal pole, δ = delta (1-4Hz), θ = theta (4-8Hz), α = alpha (8-12 Hz), β1 = low beta (12-20 Hz), β2 = high beta (20-28 Hz), γ = gamma (>28 Hz).

Supplemental discussion

Generalizability of a single case

As this study is based on a single case, as opposed to a group study or a group of case studies, we must carefully consider the extent to which it is reasonable to generalize its results. In principle the study of a single case can illuminate a condition as a whole, provided the study is appropriately controlled so as to contrast only changes in the relevant condition, and that the individual case is a typical one. As the present study was tightly-controlled, the pertinent issue is how typical the subject's tinnitus was of tinnitus as a whole. The presence of hearing loss as an underlying cause, the frequency match of 5 kHz, and the tonal quality are all typical characteristics in tinnitus patients (see the summary of 1,630 consecutive clinical tinnitus cases at www.tinnitusarchive.org/dataSets). There is a well-recognised population of tinnitus patients with normal pure-tone audiograms, unlike the severe hearing loss in our subject, however it is uncertain whether tinnitus can exist without *any* hearing loss, as studies investigating these populations have found evidence of subclinical cochlear damage [S1, S2]. Regarding the typicality of the neurophysiological results, although the present findings feature an unprecedented level of detail, a comparison can still be made between some of the simpler results and equivalent findings in the literature. Auditory cortex delta/theta power was reduced with tinnitus suppression, in line with what has been consistently observed in previous studies [S3–S6], including the one previous human intracranial study of tinnitus [S7]. The next most striking finding was the change in gamma (and beta) power that was less widespread than delta/theta power changes. Several resting-state studies of tinnitus have found gamma power increases in auditory cortex [S6, S8–S11], with no studies finding resting-state gamma decreases, and one of these that compared spatial extent found that delta was more widespread than gamma power change [S6]. A previous group of case studies found that transient changes in tinnitus intensity were accompanied by significant auditory cortex gamma power changes in most individuals (low signal to noise ratio in the remainder could explain the absence of such changes in others), but the relationship between tinnitus intensity and gamma power could be positive or inverse [S4]. Thus the observation of alterations in gamma power associated with fluctuations in tinnitus intensity is typical (as is a resting-state increase in gamma power in tinnitus) but a typical direction of such fluctuation-linked changes, at the individual level, appears not to exist. Our detailed exposition of the decoupling that occurs in auditory areas, during tinnitus suppression, between low and high frequencies is consistent with studies showing increases in the temporal association between these frequencies in tinnitus vs. control groups [S8, S12] and in baseline vs. electrically-suppressed tinnitus [S7]. Finally, the anatomical configuration of our observed tinnitus perception network (TPN), and (especially) the distribution of cross-frequency interaction hubs, closely matches the layout of a 'tinnitus core' network that has recently been proposed based on a synthesis of largely indirect evidence from studies of tinnitus and normal perception [S13]. In summary, there is strong concordance between the present findings with both existing experimental evidence and theoretical models.

Relevance of tinnitus 'subtypes'

We must also consider whether some of our findings may be specific to a particular tinnitus subtype as opposed to all the condition in general. Tinnitus varies over a large number of phenomenological dimensions, and a body of research has investigated the neurophysiological correlates of many of these factors, in the form of abnormalities of resting-state cortical oscillations. Notably, most of these differences were found only in areas separate to those in which we found tinnitus-linked changes. For many comparisons, only differences in right hemisphere oscillatory activity were observed, but given our subject's left-handedness we should not assume that these are irrelevant to the present findings. Higher levels of tinnitus distress were only associated with alpha band changes, in the form of increased power in PHC [S14]. Our subject had only mild tinnitus distress, but nonetheless had tinnitus-linked PHC alpha changes, though it remains possible that the extent or pattern of these might have been different had he been more distressed. Narrow-band noise tinnitus was found to be associated with increased gamma power in right PHC [S15] compared to pure tone tinnitus as our subject had. Thus, if anything, our findings might have underestimated gamma changes in PHC. Our subject had bilateral tinnitus, but it has been found that unilateral tinnitus is associated with increased high-frequency power in right auditory cortex, IPC and PHC [S16]. Thus, again,

had our subject had unilateral tinnitus we might have expected greater power changes in these regions. Long-duration tinnitus (as our subject had) was found to associate with generalized increases in long-range theta and alpha connectivity [S17], whereas we observed tinnitus-linked connectivity changes only in the delta band. Long duration of tinnitus was also associated with increased gamma power in right auditory cortex [S17], and thus could also have increased the magnitude of gamma power changes we observed, but as gamma changes are so ubiquitous in tinnitus it is unlikely to be a sole explanation. Other differences studied include sex [S18], associated hyperacusis [S19], and age of onset [S20], though the correlates of these factors did not overlap with our present findings. In summary, available evidence suggests that very few of our findings are likely to have been influenced by the subject's specific tinnitus subtype, in contrast to the strong concordance between our findings and generic neural signatures of tinnitus discussed in the main report.

Supplemental experimental procedures

Subject

The subject was a 50 year-old left-handed male (left-sided language dominance per Wada test) with moderate to severe bilateral high-frequency hearing loss resulting from noise trauma due to recreational handgun firing. He had chronic bilateral tonal tinnitus of approximately 15 years duration, which had begun gradually and then remained perceptually constant for most of that time, with a best frequency match of 5 kHz. His tinnitus level was matched to a 24 dB HL 500 Hz tone, and was masked by a speech-spectrum shaped noise at 54 dB HL to the left ear and 60 dB HL to the right ear. His audiometric data are illustrated in Figure S1A. Distortion product otoacoustic emissions (DPOAE) response to noise floor ratios were 2, 2, 2, 0 and 3 dB (right) and 10, 9, 0, 0 and 3 dB (left) at 1.5, 2, 3, 4 and 6 kHz respective; in all instances these indicated 'fail' (abnormal) results. His Tinnitus Handicap Inventory (THI) score was 20, indicating mild severity tinnitus. He had suffered from typical 'temporal lobe' type complex partial seizures since postnatal day 10 (which did not include any auditory aura or relate to any changes in his tinnitus), and we studied him during a 2 week period of invasive electrode monitoring performed prior to resection of the presumed focus of seizure origin. He did not suffer any seizures during this period, and subsequently a subtle congenital cortical malformation in the left posterior inferior temporal lobe was resected. The malformed area did not show any altered neural activity in relation to changes in his tinnitus, but the area of tissue resection included the posterior end of parahippocampal cortex (PHC) which did show tinnitus-related neural activity changes. His anti-epileptic regimen consisted of high-dose levetiracetam monotherapy. This was discontinued for clinical reasons for the second week of the invasive monitoring period, during which the experiments took place. The patient was not taking any other medications during the experiments reported here. The resection resulted in significantly reduced seizure frequency, and complete and sustained cessation of tinnitus in the right (contralateral) ear, with no change to tinnitus in his left ear. Previously, tinnitus laterality has been associated with PHC gamma band activity, with stronger power in the PHC contralateral to the tinnitus ear [S21], and disruption of activity in PHC has been associated with success of auditory cortex stimulation to treat tinnitus [S22]. Thus our finding of tinnitus elimination through disruption of PHC lends further evidence to its crucial importance in tinnitus. The study was approved by the University of Iowa Institutional Review Board, and written informed consent was obtained from the subject prior to any experimentation.

Experimental paradigm

The basis of the experiment was inducing a temporary suppression of perceived tinnitus loudness, using a technique called residual inhibition (RI) [S4, S23]. The phenomenon of RI is a reduction in tinnitus loudness that occurs following the presentation of an auditory stimulus and lasts beyond the end of the stimulus, typically for <30s in duration [S24]. Data recorded during this time period after the stimulus has finished, but before tinnitus has returned to normal, can be contrasted with data recorded during a period of normal tinnitus intensity (ideally recorded during the equivalent time period following a similar or identical masker stimulus but not resulting in RI) to identify only neural activity tightly yoked to tinnitus in real-time. On each of two days, we presented 30 repetitions of a 30 s broadband (0 to 22050 Hz) white noise masker stimulus, via insert earphones (ER4B; Etymotic Research, Elk Grove Village, IL), through molds fitted to the subject's ear, to both ears at the loudest comfortable volume. After each masker finished, the subject gave 4 ratings of his current perceived tinnitus loudness on a 5-point integer scale ranging from -2 (very quiet) through 0 (normal) to +2 (very loud). Ratings were prompted immediately after the end of the masker, and then after subsequent intervals of 10 s since the recording of the previous rating. The duration of the maskers, plus the number and duration of the subsequent silent periods, were determined by psychophysical testing before electrode implantation. The experimental paradigm and behavioral results are illustrated in Figure 1. Experiments were performed in a dedicated electrically shielded suite located within the Clinical Research Unit of the University of Iowa Institute for Clinical and Translational Science, and were run using a custom-written Matlab (The Mathworks Inc., Natick, MA) script using Cogent 2000 (http://www.vislab.ucl.ac.uk/cogent_2000.php), with an experimenter taking the ratings verbally from the subject and entering them into the computer using the Matlab program. The subject's full wakefulness and cooperation at all times were ensured and confirmed by the experimenter. During the three 10 s intervals between ratings that

followed each masker, the room was silent, no stimuli were presented, neither subject nor experimenter spoke or performed any actions, and the subject was instructed not to perform any mental or physical task. To keep the experiment tightly controlled, only data recorded during these 10 s silent taskless periods was analyzed in this study, except where otherwise stated. The procedure and subsequent analyses were chosen to minimise confounding factors, such as the nature, loudness or recency of the preceding auditory stimulus, and any task being performed. Importantly, changes in attention were limited, as the subject was alert and vigilant, and performed a single task of monitoring tinnitus loudness when prompted as part of the experiment. Vocal responses were brief, and occurred outside of analysed data epochs. Separately, we presented 125 repetitions of a 1 s duration 5 kHz tone, psychophysically matched to his tinnitus frequency and the loudness of a 55 dB_A tone at a normal-hearing frequency (1 kHz). A 40 Hz sinusoidal amplitude modulation was applied to the tones, in order to elicit auditory steady-state responses (ASSRs) on top of transient evoked and induced responses. Additional, broadband, auditory stimuli were each presented 50 times, and comprised click trains (0.2 ms click duration, 100 Hz and 25 Hz click rates, 200 ms duration), and consonant-vowel transitions (namely /da/, 180 ms duration).

Data acquisition
Data presented were acquired from 164 intracranial electrodes (Ad-Tech Medical Instrument, Racine, WI) that comprised 18 depth electrode contacts, in Heschl's gyrus and anterior and posterior temporal lobes in the left hemisphere plus anterior temporal lobe in the right hemisphere, and 146 subdural grid and strip electrodes (platinum-iridium disc 2.3 mm exposed diameter, embedded in a silicone membrane) covering almost all of the temporal lobe surface and adjacent paracentral, parietal and occipital cortex in the left hemisphere, plus the right temporal pole and parahippocampal gyrus. In addition, results from a depth electrode in and around the congenital cortical malformation are presented, sampling from both the lesion itself and the posterior mesial temporal lobe. Note that electrodes are numbered from 1 to 250, according to their connections with the amplifier, and there were some gaps relating to inputs that were not used. All electrodes were referenced to a common subgaleal electrode, except for analyses of long-range phase coherence (phase locking value; PLV) which were re-run using bipolar electrode pairs as described later. Signals were amplified and recorded (0.7-800 Hz bandpass, at 2034 Hz sampling rate) using a Tucker Davis Technologies (Alachua, FL) amplifier, and downsampled off-line to 1 kHz. Frequencies containing electrical noise were removed by notch filtering.

Preventing spurious correlations
We considered possible sources of spurious correlations between the signals recorded at different electrodes, and took necessary steps to eliminate these. Signal leakage in EEG/MEG scalp recordings and source reconstructions is a major problem, as the sensors are so distant from the sources that every sensor receives signal from a large expanse of brain tissue. As such, instantaneous coherence between pairs of sensors or sources is often discarded altogether [S25, S26]. However, in ECoG and depth electrode data the sensors are very close to the cortical sources from which they measure. Given a cortical thickness of under around 5mm, and generally a minimum spacing of 10mm between electrodes, even adjacent contacts should leak a maximum of 1/16 of their signal power to each other, and non-adjacent electrodes a minimum of 1/256 of their power. Therefore any spurious correlations from signal leakage should be very small in magnitude, and limited to adjacent electrodes only. In support of this, previous work has found a very rapid drop-off of spurious coherence due to volume conduction with increasing distance, with coherence values falling to 0.1 at a separation of 0.8-1.4 mm and being negligible by 3 mm [S27]. In our results, we see patterns that are incompatible with simple volume conduction, namely cross-frequency coupling changes evident strongly in certain electrodes and not at all in their neighbors, and long-range coherence changes between distant electrodes but not immediate neighbors. Nonetheless, to provide even greater robustness against spurious coherence, PLV analyses were performed using bipolar montages of adjacently located and numbered electrodes (i.e. an electrode pair was created wherever two adjacently-numbered electrodes were physically adjacent, and its time series was simply the difference of the time series from its two constituent electrodes). The other potential source of spurious correlations is signal contamination due to brain or non-brain activity recorded at the common reference electrode. Though the subgaleal reference electrode was outside the brain, we nonetheless excluded the possible influence of reference contamination on

the results obtained as follows; the raw electrode data were high-pass filtered from 200 Hz, and the covariance matrix (over electrodes) calculated. The high-pass filtering results in a covariance matrix that only represents spurious connections, as little or no true synchrony within local field potentials should exist in such a high frequency range. Conversely, any signal due to reference contamination should show an identical covariance pattern at all frequencies. A principal component analysis (PCA) was performed on this covariance matrix, which was used to construct a filter that removed the first principal component (PC), capturing any synchronous signal due to reference contamination. This filter was then applied to the full-bandwidth raw electrode data. This approach is based on previously published work [S28], and is the subject of a manuscript in preparation by members of our group. Note that this approach is designed to correct for common reference effects, rather than volume conduction. All results displayed are from analyses incorporating this 'denoising' procedure, but notably the procedure did not appreciably alter any of the final results.

Data processing

Nine frequency bands were pre-defined, as follows, to provide a trade-off between logarithmic spacing and adherence to pre-defined functional bands: delta (1-4 Hz), theta (4-8 Hz), alpha (8-12 Hz), beta1 (12-20 Hz), beta2 (20-28 Hz), gamma1 (28-44 Hz), gamma2 (44-60 Hz), gamma3 (60-92 Hz) and gamma4 (92-148 Hz). As the delta bandwidth is greater than its lowest frequency (which can compromise the quality of an analytic signal), the band was divided into two sub-bands (1-2 and 2-4 Hz), the relevant neural activity metrics were calculated (as described below) and were then averaged across the two sub-bands. Data were rejected if their power at any electrode/frequency combination exceeded a specified number of standard deviations (SD) from the mean for that electrode/frequency. For the power analysis, epochs whose log-transformed power exceeded +/- 5 SD from the mean were excluded, and for coupling analyses data points whose non-log-transformed power exceeded +/- 20 SD were excluded.

A 'tinnitus regressor' was produced, which assigned a numerical value to subjective tinnitus intensity for every epoch. This was initially calculated by averaging the numerical ratings given immediately before and after the epoch. Subsequently, to eliminate any spurious results due to the overall passing of time in the experiment, or the time elapsed since the end of the most recent masker, the tinnitus regressor was 'partialized' with respect to these factors using a process of recursive Gram-Schmidt orthogonalization implemented in SPM12 (http://www.fil.ion.ucl.ac.uk/spm/software/spm12/). The original ratings and 'partialized' ratings are shown in full in Figure 1, as blue and red markers respectively. Finally, the regressor was inverted, to reflect tinnitus *suppression* rather than tinnitus *intensity*.

For analysis of oscillatory power, a single value was obtained for each electrode/frequency/trial combination by taking the fast Fourier transform (FFT) of Hanning-windowed 1s sub-epochs and averaging across time over the epoch duration and across frequency within the specified band. Before this averaging process, spectral data were whitened (by multiplying by frequency, in order to balance the contributions of the higher and lower frequencies within each band), converted to power (by squaring the absolute value) and log-transformed. For each electrode/frequency combination, the Pearson product moment correlation coefficient (*r* value) was calculated between activity across trials and the tinnitus regressor.

For analysis of time-frequency power changes during and following presentation of noise maskers, power was calculated within the same Hanning-windowed sub-epochs and frequency bands as before, but with a moving window length of 2.5 s, covering the time period from 5 s before masker onset to 30 s after masker offset (as opposed to just the 10 s silent, taskless periods), and were not averaged over time. Thus one power value was obtained for each channel/trial/frequency/time combination. Power values were log transformed, and mean power, across time and trials within the pre-masker period, was log subtracted. These values were then averaged over electrodes comprising a pre-defined region of interest consisting of electrodes within HG and overlying HG (electrode numbers 23, 24, 61, 66, 82, 87, 161, 162, 163 and 164). Averages over trials were then taken separately for masker presentations that did and did not produce RI lasting beyond the first tinnitus rating, and multiplied by 10 to yield ERSPs as defined at the end of this section. To compare RI and non-RI trials, a two-sample T test was performed for each time-frequency point, and the results expressed as a spectrotemporal image of T scores. This image was thresholded using a non-parametric, cluster-based permutation approach [S29], treating time-

frequency points as adjacent if they differed by one time point or one frequency point (not both). A threshold of $p < 0.25$ uncorrected was applied before allocating points to clusters in each permutation, and a cluster threshold of $p < 0.05$ corrected was applied to the clusters themselves.

For analysis of local cross-frequency coupling, for each electrode/epoch combination, the time series was band-pass filtered in the frequency domain (with application of a Hanning window) and converted into a complex Hilbert time series. The Hilbert envelope was squared, log-transformed, mean-centered, normalized, and Fourier transformed again, to yield the complex spectrum of the power envelope changes of the original band-passed signal. This type of time series could thus be directly compared across frequencies. Cross-spectral density (CSD) was then calculated between each frequency pair (except between adjacent frequencies, which could be spuriously influenced by signal leakage or a variable frequency brain rhythm straddling the two bands) by averaging, over frequency, the product of the lower-frequency envelope spectrum and the complex conjugate of the higher-frequency envelope spectrum. Thus a single complex CSD was calculated for every electrode/frequency/frequency/epoch combination. This measure is similar to covariance, in that its real component *is* covariance, but also contains an imaginary component that captures time-lagged, as opposed to instantaneous, relationships. At each electrode/frequency/frequency combination, a pair of Pearson's *r* values (one real, one imaginary) was calculated between (the real and imaginary parts of) CSD and the tinnitus regressor. These two-dimensional Cartesian CSD regression coefficients were converted to polar coordinates (with theta representing CSD angle change and rho representing CSD magnitude change). Statistical testing was performed, using a permutation approach in the whole analysis space, separately on CSD magnitude and angle changes. CSD magnitude was calculated as the difference between rho calculated during the baseline tinnitus state only (only trials with a tinnitus intensity of 0), and rho calculated during a partial tinnitus suppression of 0.42 (the largest generally reached during the experiment, after partialization of the regressor). Similarly, CSD angle change was quantified as the difference in theta (constrained to the range –pi to pi) between these two states, but multiplied by the average rho of the two states (to weight angle changes by magnitude and therefore prevent instances of tiny baseline magnitudes combined with large angle changes from distorting the null distribution). In both cases, the regression was performed in Cartesian space (real and imaginary components) akin to recommended procedure for circular regression [S30], the origin (baseline state) and terminus (baseline state plus tinnitus-linked change) of the regressor were converted into polar coordinates, then paths between these points were interpolated in polar coordinates (i.e. one path for angle and one for magnitude). CSD magnitude indicates the strength of dependence between two frequency band envelopes, and CSD angle indicates the phase lag inherent in that coupling.

To analyze within-frequency long-range coupling, we calculated phase-locking value (PLV) between every possible combination of two bipolar electrode pairs (for every frequency band). This was obtained by, for each epoch, dividing the complex time series of the Hilbert-transformed raw bandpass-filtered signal by its amplitude to yield a complex time series representing phase only. For each channel pair, at each time point, the phase difference was calculated by multiplying one channel's value with the complex conjugate of the other channel's value. Phase differences were represented as vectors on the complex plane, and the vector average over time was calculated, yielding one complex PLV for each epoch for each channel pair (for each frequency). Again, for each frequency/electrode pair combination, PLV was regressed against the tinnitus regressor to yield a complex pair of 'r' values (one real, one imaginary). For bipolar electrode analyses, there is no easy interpretation of positive versus negative coherence (because some signals become inadvertently and arbitrarily inverted, depending on the positions of the underlying sources), and thus we expressed changes as the absolute change in PLV, with the putative assumption that coherence was likely reduced with tinnitus suppression.

Responses to externally presented sounds were calculated by averaging LFP waveforms across trials to yield evoked potentials (click trains and consonant-vowel stimuli), averaging after high-pass filtering from 10 Hz to yield middle-latency responses (click trains), averaging after application of a band-pass filter around 100 Hz to yield frequency-following responses (click trains), and decomposing using Morlet wavelets (click trains, consonant-vowel stimuli and amplitude modulated tones). In addition, for direct comparison with the tinnitus-linked oscillatory power analysis, the same analysis was applied to the amplitude modulated 5 kHz tones, but just taking the 0-300 ms time window, which captured the maximal response.

Statistical analysis
The basis for statistical analysis of all results presented was permutation testing. This was necessary on account of the vast data space (1,476 to 242,064 comparisons per analysis) in order to appropriately correct for multiple comparisons without making assumptions about data correlations. For each analysis 1000 permutations were performed, and in each permutation the subjective tinnitus ratings were shuffled and the analysis performed otherwise as normal. The largest absolute value occurring, anywhere in the full data space, was added to a null distribution, and the significance threshold for the actual data taken to be the 50th-highest value in the null distribution (corresponding to $p < 0.05$ corrected for the full analysis space).

Supplemental references

S1. Weisz, N., Hartmann, T., Dohrmann, K., Schlee, W., and Norena, A. (2006). High-frequency tinnitus without hearing loss does not mean absence of deafferentation. Hear. Res. *222*, 108–14.

S2. Schaette, R., and McAlpine, D. (2011). Tinnitus with a normal audiogram: physiological evidence for hidden hearing loss and computational model. J. Neurosci. *31*, 13452–7.

S3. Kahlbrock, N., and Weisz, N. (2008). Transient reduction of tinnitus intensity is marked by concomitant reductions of delta band power. BMC Biol. *6*, 4.

S4. Sedley, W., Teki, S., Kumar, S., Barnes, G. R., Bamiou, D.-E., and Griffiths, T. D. (2012). Single-subject oscillatory γ responses in tinnitus. Brain *135*, 3089–100.

S5. Adjamian, P., Sereda, M., Zobay, O., Hall, D. A, and Palmer, A. R. (2012). Neuromagnetic indicators of tinnitus and tinnitus masking in patients with and without hearing loss. J. Assoc. Res. Otolaryngol. *13*, 715–31.

S6. Tass, P. A., Adamchic, I., Freund, H.-J., von Stackelberg, T., and Hauptmann, C. (2012). Counteracting tinnitus by acoustic coordinated reset neuromodulation. Restor. Neurol. Neurosci. *30*, 137–59.

S7. De Ridder, D., van der Loo, E., Vanneste, S., Gais, S., Plazier, M., Kovacs, S., Sunaert, S., Menovsky, T., and van de Heyning, P. (2011). Theta-gamma dysrhythmia and auditory phantom perception. J. Neurosurg. *114*, 912–21.

S8. Llinás, R. R., Ribary, U., Jeanmonod, D., Kronberg, E., and Mitra, P. P. (1999). Thalamocortical dysrhythmia: A neurological and neuropsychiatric syndrome characterized by magnetoencephalography. Proc. Natl. Acad. Sci. U. S. A. *96*, 15222–7.

S9. Weisz, N., Müller, S., Schlee, W., Dohrmann, K., Hartmann, T., and Elbert, T. (2007). The neural code of auditory phantom perception. J. Neurosci. *27*, 1479–84.

S10. Ashton, H., Reid, K., Marsh, R., Johnson, I., Alter, K., and Griffiths, T. (2007). High frequency localised “hot spots” in temporal lobes of patients with intractable tinnitus: a quantitative electroencephalographic (QEEG) study. Neurosci. Lett. *426*, 23–8.

S11. Van der Loo, E., Gais, S., Congedo, M., Vanneste, S., Plazier, M., Menovsky, T., Van de Heyning, P., and de Ridder, D. (2009). Tinnitus intensity dependent gamma oscillations of the contralateral auditory cortex. PLoS One *4*, e7396.

S12. Vanneste, S., Song, J.-J., and De Ridder, D. (2013). Tinnitus and musical hallucinosis: the same but more. Neuroimage *82*, 373–83.

S13. De Ridder, D., Vanneste, S., Weisz, N., Londero, A., Schlee, W., Elgoyhen, A. B., and Langguth, B. (2013). An integrative model of auditory phantom perception: Tinnitus as a unified percept of interacting separable subnetworks. Neurosci. Biobehav. Rev. *44*, 16-32.

S14. Vanneste, S., Plazier, M., der Loo, E. Van, van de Heyning, P., Congedo, M., and de Ridder, D. (2010). The neural correlates of tinnitus-related distress. Neuroimage *52*, 470–80.

S15. Vanneste, S., Plazier, M., van der Loo, E., van de Heyning, P., and de Ridder, D. (2010). The differences in brain activity between narrow band noise and pure tone tinnitus. PLoS One *5*, e13618.

S16. Vanneste, S., Plazier, M., van der Loo, E., van de Heyning, P., and de Ridder, D. (2011). The difference between uni- and bilateral auditory phantom percept. Clin. Neurophysiol. *122*, 578–87.

S17. Vanneste, S., van de Heyning, P., and de Ridder, D. (2011). The neural network of phantom sound changes over time: a comparison between recent-onset and chronic tinnitus patients. Eur. J. Neurosci. *34*, 718–31.

S18. Vanneste, S., Joos, K., and de Ridder, D. (2012). Prefrontal cortex based sex differences in tinnitus perception: same tinnitus intensity, same tinnitus distress, different mood. PLoS One *7*, e31182.

S19. Song, J.-J., de Ridder, D., Weisz, N., Schlee, W., van de Heyning, P., and Vanneste, S. (2014). Hyperacusis-associated pathological resting-state brain oscillations in the tinnitus brain: a hyperresponsiveness network with paradoxically inactive auditory cortex. Brain Struct. Funct. *219*, 1113–28.

S20. Song, J.-J., de Ridder, D., Schlee, W., van de Heyning, P., and Vanneste, S. (2013). "Distressed aging": the differences in brain activity between early- and late-onset tinnitus. Neurobiol. Aging *34*, 1853–63.

S21. Vanneste, S., van de Heyning, P., and de Ridder, D. (2011). Contralateral parahippocampal gamma-band activity determines noise-like tinnitus laterality: a region of interest analysis. Neuroscience *199*, 481–90.

S22. De Ridder, D., and Vanneste, S. (2014). Targeting the Parahippocampal Area by Auditory Cortex Stimulation in Tinnitus. Brain Stimul. *7*, 709–717.

S23. Roberts, L. E. (2007). Residual inhibition. Prog. Brain Res. *166*, 487–95.

S24. Roberts, L. E., Moffat, G., Baumann, M., Ward, L. M., and Bosnyak, D. J. (2008). Residual inhibition functions overlap tinnitus spectra and the region of auditory threshold shift. J. Assoc. Res. Otolaryngol. *9*, 417–35.

S25. Gross, J., Baillet, S., Barnes, G. R., Henson, R. N., Hillebrand, A., Jensen, O., Jerbi, K., Litvak, V., Maess, B., Oostenveld, R., et al. (2013). Good practice for conducting and reporting MEG research. Neuroimage *65*, 349–63.

S26. Hipp, J. F., Hawellek, D. J., Corbetta, M., Siegel, M., and Engel, A. K. (2012). Large-scale cortical correlation structure of spontaneous oscillatory activity. Nat. Neurosci. *15*, 884–90.

S27. Holsheimer, J., and Feenstra, B. W. (1977). Volume conduction and EEG measurements within the brain: a quantitative approach to the influence of electrical spread on the linear relationship of activity measured at different locations. Electroencephalogr. Clin. Neurophysiol. *43*, 52–8.

S28. Mitra, P., and Bokil, H. (2007). Observed Brain Dynamics 1st ed. (Oxford: Oxford University Press).

S29. Maris, E., and Oostenveld, R. (2007). Nonparametric statistical testing of EEG- and MEG-data. J. Neurosci. Methods *164*, 177–90.

S30. Berens, P. (2009). Circstat: A MATLAB Toolbox for Circular Statistics. J. Stat. Softw. *31*.

S31. Liégeois-Chauvel, C., Musolino, A., Badier, J. M., Marquis, P., and Chauvel, P. (1994). Evoked potentials recorded from the auditory cortex in man: evaluation and topography of the middle latency components. Electroencephalogr. Clin. Neurophysiol. Potentials Sect. *92*, 204–214.
